# Genome analysis of two novel *Pseudomonas* strains exhibiting differential hypersensitivity reactions on tobacco seedlings reveals differences in nonflagellar T3SS organization and predicted effector proteins

**DOI:** 10.1002/mbo3.553

**Published:** 2018-02-21

**Authors:** Caetanie F. Tchagang, Renlin Xu, Cyr Lézin Doumbou, James T. Tambong

**Affiliations:** ^1^ Ottawa Research and Development Centre Ottawa ON Canada; ^2^ Institut des sciences de santé et de la vie Collège La Cité Ottawa ON Canada

**Keywords:** digital DNA‐DNA hybridization, genome sequencing, MuMmer average nucleotide identity, *Pseudomonas*, T1SS, T3SS

## Abstract

Multilocus sequence analysis (MLSA) of two new biological control strains (S1E40 and S3E12) of *Pseudomonas* was performed to assess their taxonomic position relative to close lineages, and comparative genomics employed to investigate whether these strains differ in key genetic features involved in hypersensitivity responses (HRs). Strain S3E12, at high concentration, incites HRs on tobacco and corn plantlets while S1E40 does not. Phylogenies based on individual genes and 16S rRNA‐*gyr*B‐*rpo*B‐*rpo*D concatenated sequence data show strains S1E40 and S3E12 clustering in distinct groups. Strain S3E12 consistently clustered with *Pseudomonas marginalis*, a bacterium causing soft rots on plant tissues. MLSA data suggest that strains S1E40 and S3E12 are novel genotypes. This is consistent with the data of genome‐based DNA‐DNA homology values that are below the proposed cutoff species boundary. Comparative genomics analysis of the two strains revealed major differences in the type III secretion systems (T3SS) as well as the predicted T3SS secreted effector proteins (T3Es). One nonflagellar (NF‐T3SS) and two flagellar T3SSs (F‐T3SS) clusters were identified in both strains. While F‐T3SS clusters in both strains were relatively conserved, the NF‐T3SS clusters differed in the number of core components present. The predicted T3Es also differed in the type and number of CDSs with both strains having unique predicted protease‐related effectors. In addition, the T1SS organization of the S3E12 genome has protein‐coding sequences (CDSs) encoding for key factors such as T1SS secreted agglutinin repeats‐toxins (a group of cytolysins and cytotoxins), a membrane fusion protein (LapC), a T1SS ATPase of LssB family (LapB), and T1SS‐associated transglutaminase‐like cysteine proteinase (LapP). In contrast, strain S1E40 has all CDSs for the seven‐gene operon (*pel*A‐*pel*G) required for Pel biosynthesis but not S3E12, suggesting that biofilm formation in these strains is modulated differently. The data presented here provide an insight of the genome organization of these two phytobacterial strains.

## 1 INTRODUCTION

Species of the genus *Pseudomonas* are aerobic Gram‐negative bacteria of the *Pseudomonadaceae* family. There are 144 validly described species divided into different groups (Garrido‐Sanz et al., 2016; Gomila, Peña, Mulet, Lalucat, & García‐Valdés, 2015). They are ubiquitous in the rhizosphere of plants, water, and soil environments. This genus includes bacterial species of environmental significance such as phytopathogens, biological control agents, plant growth promoters, and xenobiotic degraders (Johnsen, Andersen, & Jacobsen, 1996; Palleroni, 2005; Pierson & Pierson, 2010; Tambong, Xu, & Bromfield, 2017). The fluorescent pseudomonads are uniquely capable of synthesizing many metabolites that play a role in maintaining soil health leading to bioprotection of crops against pathogens (Garrido‐Sanz et al., 2016; Gaur, Shani, Kawaljeet, Rossi, & Posada, 2004; Mazzola, Cook, Thomashow, Weller, & Pierson, 1992). Because of this plant bioprotection characteristic, members of this genus are routinely being isolated from various environments and evaluated for antagonistic activity against major plant fungal plant pathogens.

In 2015, two *Pseudomonas* strains (S1E40 and S3E12), isolated from corn roots inoculated with a 10% soil suspension under controlled conditions, were evaluated for biological control and induction of hypersensitivity to tobacco and corn seedlings. Both strains exhibited potent *in vitro* antagonistic activity against a major fungal plant pathogen, *Rhizoctonia solani*. However, these novel strains exhibited differential hypersensitivity reactions (HRs) on tobacco and corn seedlings. At high bacterial concentrations, strain S3E12 induced severe HRs on tobacco and corn seedlings, but strain S1E40 did not. This differential response of corn and tobacco to inoculations with S1E40 and S3E12 is an interesting phenomenon worthy to be studied.

Microbial pathogens/beneficial strains interact with plant hosts by a complex multistep process (Abby et al., 2016; Poueymiro et al., 2009; Raymond et al., 2013). Bacteria are known to have wide variety of biotic associations with plant hosts. These include the formation of biofilms and mutualistic or pathogenic associations in which protein secretion (Abby et al., 2016; Martinez‐Garcia, Ramos, & Rodriguez‐Palenzuela, 2015; Petnicki‐Ocwieja et al., 2002) can play a pivotal role in controlling the interactions. It is possible that the differences in the induction of HRs on tobacco and corn by S1E40 and S3E12 could be due to differences in the protein secretion systems in these strains. Therefore, it is important to identify any genetic differences between the strains in order to understand and target pathogenicity/biocontrol factors.

Next‐generation sequencing followed by analysis of entire bacterial genomes is becoming a routine tool in studying genetic differences between species, subspecies, and strains. In addition, rapid increase in genome data has expanded our knowledge of the complexity of bacterial proteins (Martinez‐Garcia et al., 2015; Tseng, Tyler, & Setubal, 2009). In addition, various computational methods (Goldberg, Rost, & Bromberg, 2016; Martinez‐Garcia et al., 2015; Wang, Zhang, Sun, & Guo, 2011) have been developed for *in silico* identification of bacterial secretion proteins in genome data. These rapid advances are valuable tools to further our understanding of phytobacterial secretion systems.

The goals of this study were (i) to sequence and assemble the genomes of *Pseudomonas* strains S1E40 and S3E12; (ii) to assess the taxonomic position of strains relative to their close lineages based on genome‐based DNA–DNA homology and multilocus sequence analysis (MLSA); (iii) to perform comparative genomics analysis of these two strains; and (iv) to use different prediction algorithms for mining the genome data of S1E40 and S3E12 for components of the type III secretion systems (T3SS) and potential effector proteins. To obtain high‐quality draft genomes from millions of Illumina MiSeq sequences (250 bp), ABySS (Simpson et al., 2009), a software based on the de Bruijn digraph approach, was used over a large k‐mer value range (50–150). Data on the assessment of the taxonomic position presented here suggest that strains S1E40 and S3E12 are unique and possibly novel species, and *in silico* T3SS analysis of the genome sequences revealed significant differences in terms of organization and predicted T3SS effector protein.

## 2 MATERIALS AND METHODS

### 2.1 Genome downloads and annotation

Eleven whole‐genome sequence (wgs) data of *Pseudomonas* strains (Table  S1) were downloaded from GenBank (Benson, Karsch‐Mizrachi, Lipman, Ostell, & Sayers, 2011) at NCBI, http://www.ncbi.nlm.nih.gov/genome/browser. This was done as previously reported (Tambong et al., 2016) using the getgbk.pl script as implemented in CMG‐Biotools (Vesth, Lagesen, Acar, & Ussery, 2013). Genome sequences were extracted from GenBank files and saved as FASTA format using the saco_convert script (Jensen & Knudsen, 1999). The wgs data of the eleven strains were submitted to the RAST (Aziz et al., 2008) and PATRIC (Wattam et al., 2014) web‐based annotation systems followed by manual curation. The wgs data downloaded were from valid‐type strains except for *Pseudomonas grimontii*,* Pseudomonas marginalis, Pseudomonas palleroniana, Pseudomonas rhodesiae,* and *Pseudomonas tolaasi*. To confirm the identity of these strains, 16S rRNA sequences were extracted from the wgs using the RNAmmer (Lagesen et al., 2007) as implemented in CMG‐Biotools (Vesth et al., 2013) and compared by BLAST and phylogenetic analyses.

### 2.2 Genome sequencing and assembly of S1E40 and S3E12

The draft genome sequences of S1E40 and S3E12 were determined by paired‐end sequencing using Illumina MiSeq technology (Génome Québec, Montreal, Canada). A total of 4,290,298 and 4,472,574 paired‐end reads, each 250 bp in length, for strain S1E40 and S3E12, respectively, were generated. The quality of the reads was checked using FastQC version 0.11.3 (Andrews, 2015). *De novo* assemblies were performed using ABySS version 1.5.2 (Simpson et al., 2009) at k‐mer values of 50–150. The best assemblies were selected based on k‐mer values with the lowest number of contigs/scaffolds. The ABySS assembly was compared to that of a user‐friendly CLC Genomics Workbench version 9.0.1 (CLC; Qiagen Inc, Canada) that has a k‐mer value limit of 64.

### 2.3 PCR amplification and DNA sequencing

16S rRNA, *gyr*B, *rpo*B, and *rpo*D genes of strains S1E40 and S3E12 were PCR‐amplified, sequenced and edited as previously reported (Tambong, Xu, Kaneza, & Nshogozabahizi, 2014; Tambong et al., 2016). The corresponding genes of closely related *Pseudomonas* species were obtained from GenBank, and the DNA sequences of each strain concatenated using Geneious 10.0 (http://www.geneious.com/). 16S rRNA‐*gyr*B‐*rpo*B‐*rpo*D maximum‐likelihood phylogenetic tree of *Pseudomonas* strains S1E40, S3E12, and 11 closely related known lineages was implemented using MEGA7 (Kumar, Stecher, & Tamura, 2016) with Kimura‐2‐parameter substitution model and 1,000 bootstrap values.

### 2.4 DNA–DNA analyses of genomes

Genome‐sequence‐based digital DNA–DNA hybridization (dDDH; Meier‐Kolthoff, Auch, Klenk, & Goker, 2013) and MUMmer‐based average nucleotide identity (ANIm; Kurtz et al., 2004) were employed to assess the taxonomic position of strains S1E40 and S3E12 relative to the closest taxa. The dDDH values were calculated using the genome‐to‐genome distance calculator (GGDC) version 2.1 (http://ggdc.dsmz.de; Meier‐Kolthoff et al., 2013). ANIm similarity values were computed as described by Kurtz et al. (Kurtz et al., 2004) and implemented in JSpecies (Richter & Rossello‐Mora, 2009).

### 2.5 Proteome comparisons

The comparison of proteomes of strains S1E40 and S3E12 was implemented using PATRIC (Wattam et al., 2014) and CMG‐Biotools (Vesth et al., 2013). PATRIC was executed using default parameters. For CMG‐Biotools, a blastmatrix was generated using an XML formatted input file created by makebmdest (Vesth et al., 2013). A pairwise proteome comparison using BLAST (Binnewies, Hallin, Staerfeldt, & Ussery, 2005) was used to generate a BLAST matrix. Protein sequences were compared to each other. Two sequences are similar and collected in the same “protein family” if the BLAST hit had at least 50% identical matches in the alignment and the length of the alignment is 50% of the longest gene in the comparison. For the comparison of two genomes, the single linkage is used to build protein families. Paralogs within a proteome are also evaluated and outputted at the bottom row of the matrix. Analysis of orthologous clusters was also performed using the FastOrtho (http://enews.patricbrc.org/fastortho/), a faster reimplementation of OrthoMCL (Li, Stoeckert, & Roos, 2003) with default parameters (e‐value of 1e^−5^ and inflation value of 1.5).

### 2.6 Identification of T3SS and effector proteins

T346Hunter (Martinez‐Garcia et al., 2015) was used to identify T3SS clusters by performing BLASTp (Altschul, Gish, Miller, Myers, & Lipman, 1990) and HMMER3 (Eddy, 2011) searches and genome regions containing genes of high homology (E ≤ 0.0005) with at least four core components of T3SS (Abby & Rocha, 2012) and spanning a size of up to 70 kb were outputted as flagellar‐related and nonflagellar T3SS (NF‐T3SS). T346Hunter uses Glimmer v3.02 (Delcher, Bratke, Powers, & Salzberg, 2007) to predict open reading frames in the genome sequences of S1E40 and S3E12; and the R package genoPlotR (Guy, Kultima, & Andersson, 2010) was used to produce the gene maps.

PATRIC (Wattam et al., 2014) annotated protein‐coding sequences where scanned for T3SS effectors using the T3SS effector prediction tool (Lower & Schneider, 2009) based on the neural network method with a default threshold of 0.4. The predicted effectors were then screened for putative effector proteins using the BPBAac effector prediction tool (Wang et al., 2011) that used a *Ralstonia solanceurum* training dataset for validation (https://biocomputer.bio.cuhk.edu.hk/T3DB/BPBAac.php). Two proteins annotated by PATRIC (Wattam et al., 2014) as potential effectors and confirmed using the prediction tool of Lower and Schneider (2009) were also included. BLASTp search with a cutoff e‐value of 10^−5^ was performed to determine predicted T3SS homolog effector proteins between S1E40 and S3E12.

### 2.7 Hypersensitivity test and growth in artificial minimal medium

Hypersensitivity study was conducted as previously reported (Shulte & Bonas, 1992) using 10^8^ colony forming units of each of the bacterial strains. Plants were observed daily for HR response. Also, the bacterial strains were inoculated in M9 minimal (M9M) medium, a nutrient‐poor medium reported to mimic nutrient availability in plant cells. The growth of strains S1E40 and S3E12 was monitored in liquid M9M medium with 10 mmol/L fructose (28°C, 235 rpm) as previously described (Shulte & Bonas, 1992). The optical density at 595 nm of aliquots was measured, in triplicates, at 24‐hr intervals using the BMG FLUOstar OPTIMA microplate reader (BMG, Canada). At the end of the 6‐day study, only strain S3E12 exhibited adequate growth. The cells of S3E12 were harvested for RNA processing and gene expression to confirm the viability. S3E12 cells were harvested by centrifugation at 10,000 rpm for 6 min, and total RNA was extracted using the NucleoSpin RNAII Kit (Clontech, Canada) following the manufacturer's instructions. Total RNA was eluted from the column with 60 μl of RNase‐free water by centrifugation at 11,000 × *g* for 1 min. The eluted total RNA was treated twice with DNA‐free Kit (Life Technologies, Canada) to remove DNA contaminations following the manufacturer's instructions. The RNA concentration was checked by NanoDrop 1000 and the quality of total RNA confirmed using Agilent 2000 BioAnalyzer (Agilent, Canada).

First‐strand cDNA synthesis was performed using the RNA‐to‐cDNA EcoDry Premix (Oligo dT) kit (Clontech, CA) with random hexamers according to the manufacturer's instructions. SyBr real‐time PCR assay was performed on the purified total RNA to check whether two targeted potential T3SS effectors and an effector‐binding protein are expressed in M9M medium reported to mimic plant cell nutrients. Specific primers, CandHop‐1F/CandHop‐330R, HopPmaJ‐1F/ HopPmaJ‐340R, and Binding‐1F/Binding‐480R (Table  S1), were designed, and simplex real‐time PCR was performed as previously reported (Tambong, 2015) using SsoFast EvaGreen Supermix kit (Bio‐RAD, Canada). One microliter of genomic DNA (1 ng/μl) from strain S3E12 was used as positive control; and 1 μl of total RNA and PCR water was used as negative controls (1 ng/μl). Melting curve analyses and agarose gel (1.8%) electrophoresis were run to confirm specificity.

## 3 RESULTS

### 3.1 Characterization of strains using MLSA

**Figure 1:**
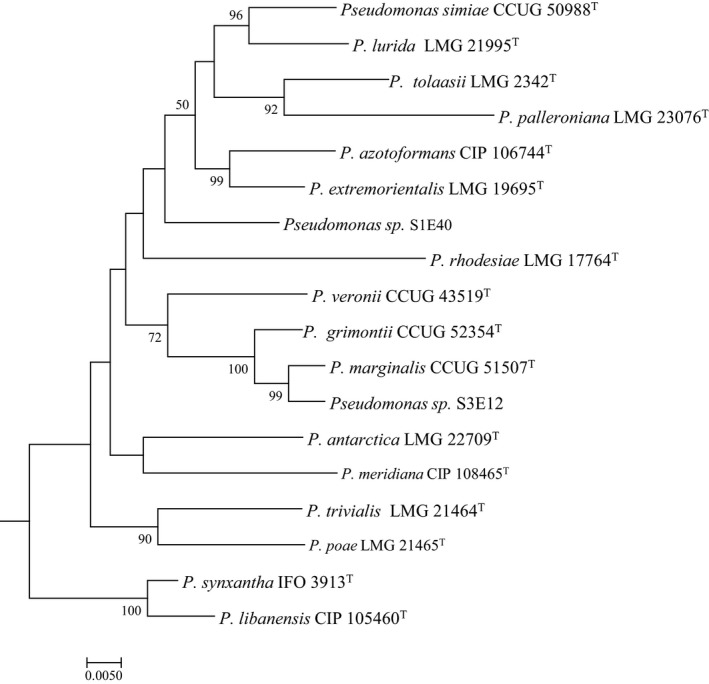
16S rRNA–gyrB–rpoB–rpoD (about 3,800 bp) concatenated tree inferred by maximum likelihood with *Pseudomonas* sp. strains S1E40 and S3E12 and type strains of closely related lineages using the general time reversible substitution (GTR). GRT substitution model was used with 1,000 bootstrap replicates. The subtree is rooted using *Pseudomonas aeruginosa* LMG 1242^T^ (outgroup)

BLAST and phylogenetic analyses were performed using four genes (16S rRNA, *gyr*B, *rpo*B, and *rpo*D) to determine the taxonomic position of S1E40 and S3E12. ML trees generated from individual gene sequences (Figure S1) and concatenated phylogeneteic trees (Figure 1) show strains S1E40 and S3E12 clustering in distinct groups. *Pseudomonas* sp. S1E40 was found to be closely affiliated to seven validly described *Pseudomonas* species while *Pseudomonas* sp. S3E12 could be grouped with four *Pseudomonas* species (Figure 1). The two new strains clustered distinctly within these subgroups. Strain S3E12 consistently clustered with *P. marginalis, P. grimontii,* and *P. veronii* (Figure 1).

### 3.2 Genome assembly statistics

**Figure 2:**
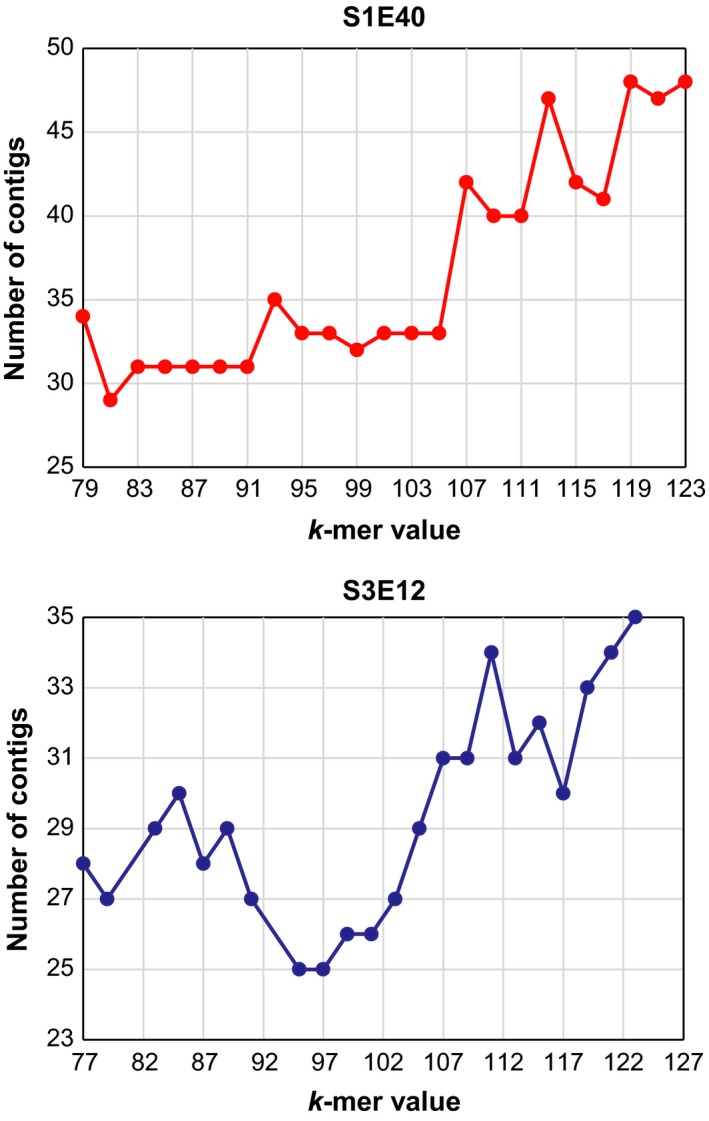
Line plots showing the influence of k‐mer values on the number of contigs obtained using ABySS assembler on Illumina MiSeq data sets for *Pseudomonas* sp. strains S1E40 (a) and S3E12 (b). The best k‐mer values (fewest number of contigs) for *Pseudomonas* strains S1E40 and S3E12 were 85 and 95, respectively

Figure 2 shows the variation of number of contigs with change in k‐mer values for S1E40 and S3E12. The lowest number of contigs obtained for S1E40 was at k‐mer = 81, while a k‐mer of 95 was observed for strain S3E12. Even though k‐mer = 81 (S1E40) gave the lowest number of contigs, it was not considered the best as some of the scaffolds were the result of merging contigs using more than 100 Ns. K‐mer values of 85 and 95 gave the best assemblies, producing 49 and 38 scaffolds after the discard of scaffolds with length <300 bp for strains S1E40 and S3E12, respectively. The total size of the draft genomes is 6,984,066 bp (minimum, 402 bp; maximum, 834,588 bp; N_50_, 206,686 bp) and 7,062,659 bp (minimum, 303 bp; maximum, 935,416 bp; N_50_, 420,043 bp) for S1E40 and S3E12, respectively. The G+C contents of the draft genomes are 61.6% and 60.9%, with overall estimated coverage of 153 ×  and 158 ×  for strains S1E40 and S3E12, respectively.

### 3.3 Genome‐based DNA–DNA homology analysis

Genome similarity analysis using dDDH and ANIm showed values ranging from 22.0% to 55.90% and 82.21%–90.44%, respectively (Table 1). *Pseudomonas* sp. S3E12 exhibited the highest dDDH homology (53.20%) with *P. grimontii* BS2976 (FNKM00000000), while strain S1E40 had the highest dDDH value (37.0%) with strain S3E12. Highest dDDH value (55.90%) is between *Pseudomonas extremeorientalis* LMG 19695^T^ and *Pseudomonas azotoformans* LMG 21611^T^. Lowest dDDH value (22.0%) is between *P. marginalis* ICMP 11289 (LKGX00000000) and *P. palleroniana* BS3265 (FNUA00000000). Similar trend was observed for ANIm values (species‐level cutoff = 95%) (Table 1). All the dDDH (cutoff = 70%) and ANIm (cutoff = 95%) values are below the proposed species boundary cutoff. Genome‐based DNA–DNA homology (dDDH and ANIm) values of strains S1E40 and S3E12 and the close *Pseudomonas* species were below the proposed species boundary cutoff, suggesting potential novel genotypes.

**Table 1:** Chromosomal genome similarities between the new *Pseudomonas* strains (S1E40 and S3E12) and known close relatives based on genome‐to‐genome digital DNA–DNA hybridization (lower diagonal) and MuMmer‐based average nucleotide identity (upper diagonal)

	Reference genome	1	2	3	4	5	6	7	8	9	10	11
1.	*Pseudomonas tolaasii* 6264 (AKYY00000000)	100	82.21%	84.70%	88.80%	87.61%	88.47%	88.51%	88.08%	88.09%	88.67%	88.62%
2.	*Pseudomonas palleroniana* (FNUA00000000)	32.30%	100	84.51%	88.43%	87.23%	88.08%	88.99%	89.22%	88.30%	88.51%	89.74%
3.	*Pseudomonas marginalis* ICMP 11289 (LKGX00000000)	22.70%	22.00%	100	84.7	84.50%	84.70%	84.70%	84.53%	84.53%	85.00%	84.70%
4.	*Pseudomonas azotoformans* LMG 21611^T^ (MDDQ00000000)	33.80%	32.90%	22.40%	100	87.85%	88.78%	90.44%	94.36%	89.31%	89.30%	89.25%
5.	*Pseudomonas rhodesiae* FF9 (CCYI00000000)	31.50%	30.50%	22.10%	32.30%	100	87.88%	87.60%	87.82%	88.10%	88.31%	88.08%
6.	*Pseudomonas veronii* DSM 11331^T^ (JYLL00000000)	33.60%	32.80%	22.50%	34.60%	32.20%	100	88.58%	88.70%	89.28%	89.24%	89.16%
7.	*Pseudomonas simiae* CCUG 50988^T^ (MDFH00000000)	33.40%	32.50%	22.40%	39.90%	31.50%	34.0%	100	90.40%	88.99%	89.18%	89.05%
8.	*Pseudomonas extremorientalis* LMG 19695^T^ (MDGK00000000)	34.00%	32.60%	22.20%	55.90%	32.20%	34.50%	39.60%	100	89.22%	94.10%	89.74%
9.	*Pseudomonas grimontii* BS2976 (FNKM00000000)	33.80%	33.40%	22.40%	35.90%	32.80%	36.00%	35.30%	35.90%	100	88.64%	88.50%
10.	*Pseudomonas* sp. S3E12 (MBDT00000000)	33.70%	33.00%	22.40%	36.10%	33.10%	35.40%	35.10%	35.80%	53.20%	100	89.60%
11.	*Pseudomonas* sp. S1E40 (MAUE00000000)	33.70%	32.80%	22.50%	35.70%	32.50%	35.40%	35.10%	35.80%	36.80%	37.00%	100

dDDH values were computed using the program GGDC 2.1 (Meier‐Kolthoff et al., 2013), and the model confidence intervals are shown in square brackets. ANIm values were computed in Jspecies program (Richter & Rossello‐Mora, 2009).

### 3.4 Comparative genomics of strains S1E40 and S3E12

**Figure 3:**
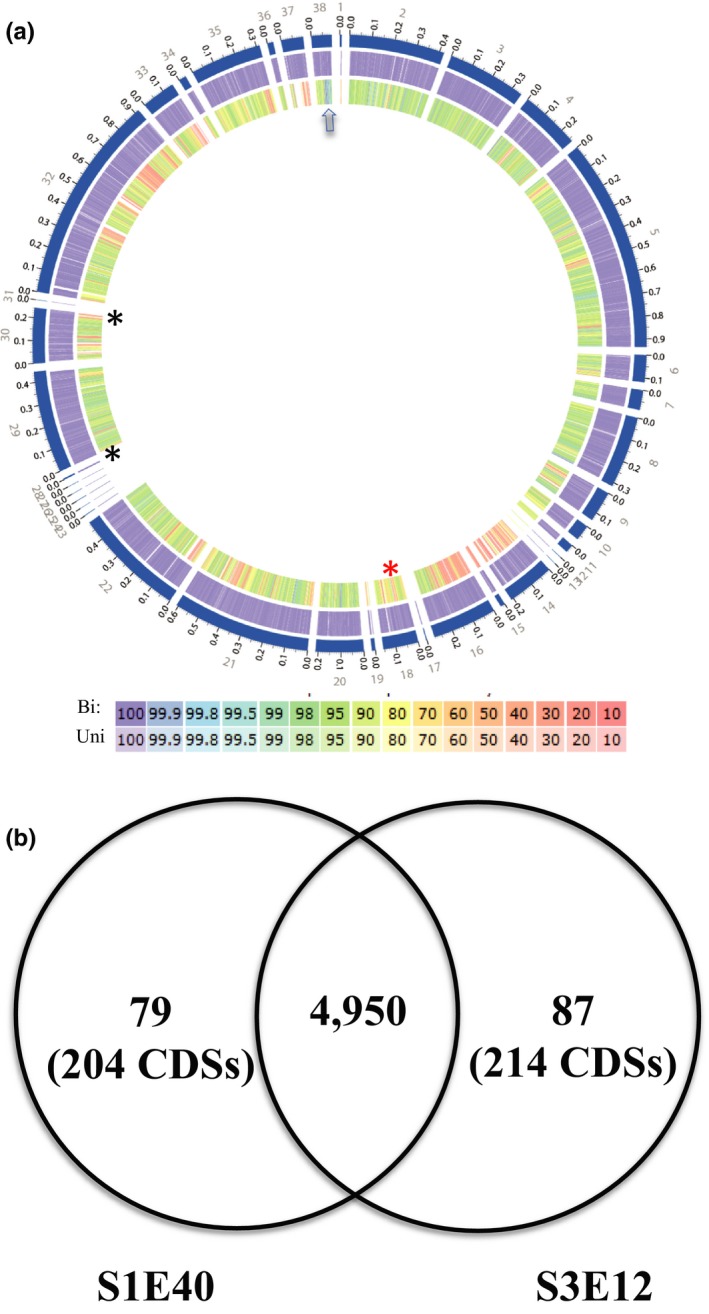
(a) Proteome comparison of genomes of *Pseudomonas* sp. S1E40 and S3E12. The outermost circle (circle 1) represents the scale (Mb) of the chromosomal (blue) of strain S3E12. Circle 2, the chromosomal protein‐coding sequences of strain S3E12 as references; circle 3, protein‐coding sequences of strain S3E40, and (b) Venn diagram showing orthologous gene groupings clustered using FastOrthoMCL. Bi, Bidirectional best hit; Uni, unidirectional best hit. Blue arrow depicts group of proteins, for example, ribosomal proteins S12p, S7p, L4p with high homology in both genomes; black asterisks show the type I secretion system for aggregation and T1SS secreted agglutinin RTX toxin proteins present in S3E12 but not S1E40; red asterisk shows location of alpha‐L‐Rha alpha‐1,3‐L‐rhamnosyltransferase in S3E12 but absent in S1E40. CDS, protein‐coding sequences

Pairwise proteome comparisons using the BLAST matrix (Vesth et al., 2013) between the genomes showed similarity ranging from 40.6% to 78.3% (Figure S2 ). Consistent with dDDH and ANIm results, strain S3E12 had the highest proteome similarity (67.7%) with *P. grimontii* BS2976 (Figure S2), while strain S1E40 exhibited the highest proteome similarity (69.2%) with *P. extremorientalis* LMG 19695^T^ (Figure S2). An overview of the conserved and specific genomic regions are shown in Figure 3a. Conserved CDSs, for example, ribosomal proteins (S12p, S7p, L4p …etc) with high homology in both genomes are depicted by a blue arrow while the square bracket and asterisks show some of the subtle CDSs present only in strain S3E12 but not in strain S1E40 (Figure 3a). These include the type I secretion system for aggregation, T1SS secreted agglutinin RTX toxin proteins, and alpha‐L‐Rha alpha‐1,3‐L‐rhamnosyltransferase that could be partly involved in the induction of hypersensitivity reaction on tobacco and corn seedlings by strain S3E12.

To identify conserved and subspecies‐specific CDSs, pan‐genome analyses including orthologous group classification and orthologous relationship were performed. Orthologous relationships were determined using the FastOrtho method. Strains S1E40 and S3E12 share 4,950 orthologous gene clusters (Figure 3b) with the former having 79 unique clusters, while S3E12 had 87 unique clusters.

**Figure 4:**
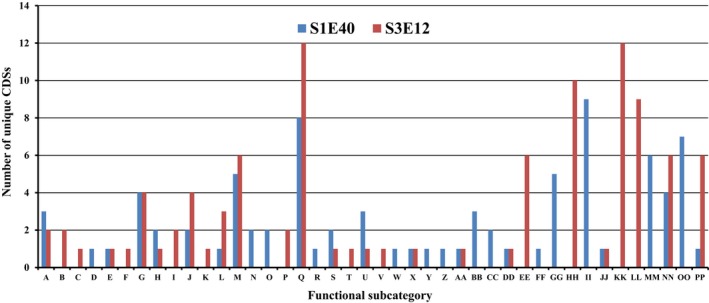
Number of unique CDSs identified using RAST seed (Aziz et al., 2008) in the draft genomes of *Pseudomonas* sp. strains S1E40 and S3E12 in 42 functional subcategories. The 42 subcategories are arginine; urea cycle, polyamines (A); aromatic amino acids and derivatives (B); branched‐chain amino acids (C); histidine metabolism (D); lysine, threonine, methionine, and cysteine (E); proline and 4‐hydroxyproline (F); central carbohydrate metabolism (G); di‐ and oligosaccharides (H); fermentation (I); monosaccharides (J); one‐carbon metabolism (K); organic acids (L); capsular and extracellular polysaccharides (M); Gram‐negative cell wall components (N); cytochrome biogenesis (O); DNA metabolism (P); clustering‐based subsystems (Q); coenzyme A (R); riboflavin, FMN, FAD (S); tetrapyrroles (T); DNA repair (U); DNA replication (V); dormancy and sporulation (W); fatty acids (X); isoprenoids (Y); phospholipids (Z); siderophores (AA); iron acquisition and metabolism‐no subcategory (BB); ABC transporters (CC); cation transporters (DD); protein secretion system, type I (EE); protein secretion system, type V (FF); protein secretion system, type VII (chaperone/usher pathway, CU) (GG); metabolism of central aromatic intermediates (HH); alkylphosphonate utilization (II); bacterial chemotaxis (JJ); denitrification (KK); nitrogen metabolism‐no subcategory (LL); phages, prophages (MM); capsular and extracellular polysaccharides (NN); quorum sensing and biofilm formation (OO); and programmed cell death and toxin–antitoxin systems (PP)

Figure 4 summarizes some of the unique functional CDSs identified in the two genomes based on RAST (Aziz et al., 2008). Strain S3E12 has unique CDSs involved in DNA metabolism and the metabolism of aromatic amino acids and derivatives, branched‐chain amino acids, proline and 4‐hydroxyproline, and one‐carbon metabolism (Figure 4). In addition, strain S3E12 has type I protein secretion system and denitrification CDSs that are not present in strain S1E40 as well as a disproportionately high number of unique CDSs implicated in programmed cell death and toxin–antitoxin systems (Figure 4). Strain S3E12 has more elaborate toxin–antitoxin systems that include VapC and HigB toxin proteins and antitoxin YgiT and RelB/StbD replicon proteins. In contrast, strain S1E40 has toxin–antitoxin replicon stabilization systems that involve ParE toxin protein (dataset 1). Strain S1E40 has unique CDSs involved in alkylphosphonate utilization, histidine degradation, cytochrome biogenesis, and phages and prophages. Unique quorum sensing and biofilm formation CDSs were also identified in strain S1E40 (Figure 4). For example, the complete *pel* gene cluster implicated in initiating and maintaining interactions between bacterial cells is identified in strain S1E40 but absent in S3E12 (Figure 4).

### 3.5 Prediction of T3SS and effector proteins in genome data

**Figure 5:**
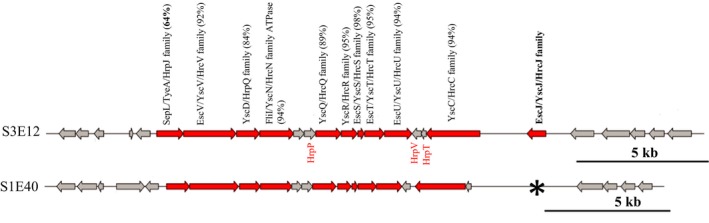
Organization of the nonflagellar type III secretion system in the genome data of *Pseudomonas* strains S3E12 and S1E40 cluster identified using T346Hunter prediction tool (Martinez‐Garcia et al., 2015). Strain S1E40 does not seem to have the T3SS protein, HrcJ, within this cluster. Also, note the low sequence homology (64%) of the type III secretion system gatekeeper, HrpJ, between strains S1E40 and S3E12. The location of HrpP, HrpV, and HrpT genes (in red) is based on annotation using PATRIC (2014). *The missing hrcJ gene

The identification and localization of T3SS clusters in the genomes of S1E40 and S3E12 were performed using T346Hunter (Martinez‐Garcia et al., 2015). One nonflagellar and two flagellar‐based T3SS clusters were identified in both genomes. The nonflagellar (NF‐T3SS) cluster (Figure 5) showed differences in the gene maps. Strain S3E12 exhibited all the nine expected core components (100%) of nonflagellar T3SS while S1E40 had only eight core components (88.9%). The T3SS genes in this cluster showed sequence similarities of 64%–98%. The lowest homology of 64% is for the protein SepL/TyeA/HrpJ family, the T3SS gatekeeper (Figure 5). Also within this cluster, the protein EscJ/YscJ/HrcJ family was identified only in strain S3E12.

One of the two clusters of flagella T3SSs revealed the presence of nine genes with only two of the expected core components for each of the bacterial strains. The third cluster comprised of 34 or 33 genes with nine of the expected core components for S1E40 or S3E12, respectively. Sequence homology of the flagellar T3SS genes was relatively conserved with similarity levels between 93 and 98%.

Based on the prediction tool of Lower and Schneider (2009), 567 and 565 potential effector proteins were identified for strains S3E12 and S1E40, respectively. These effector proteins were used as input data for BPBAac prediction tool (Wang et al., 2011), a tool validated using the effector dataset of the plant pathogen *Ralstonia solanacearum*, leading to a reduced list of 32 and 29 potential putative effector proteins for strains S1E40 and S3E12, respectively (Table 2). Two proteins annotated as effectors by PATRIC (Wattam et al., 2014) and confirmed by the tool of Lower and Schneider (2009) were added to the list of BPBAac (Table 2). BLASTp (e‐value of 10–5) comparison between S3E12 (query) and S1E40 (database) showed similarity values of 90%–99% for 11 effector proteins. Low similarity values of 31%, 36%, 39%, and 52% were observed for four protein sequences, while 16 effector proteins predicted in S3E12 did not have a homolog among the 34 potential effector proteins of strain S1E40 (Table 2). Blast results of the 31 and 34 to a customized database of 756 effectors from pathovars of *Pseudomonas syringae* identified a HopB1‐like effector (37% homology) of *P. s. *pv.* aceris* strain M302273 in each of the strains. In addition, strain S3E12 has a HrpK‐like effector protein with low homology to that of *Pseudomonas syringae* pv*. castaneae*.

**Table 2:** Predicted type III secreted effector proteins identified in the hypersensitivity responses‐inducing strain S3E12 and similarity values of homologs in strain S1E40^a^

PATRIC gene identifier	Protein	Lower score	BPBAac score	Homolog in S1E40	
				Gene id	Similarity (%)
fig|1873126.6.peg.247	Outer membrane protein ImpK/VasF, OmpA/MotB domain	0.47	0.53	fig|1869229.4.1934	98
fig|1873126.6.peg.741	Poly(A) polymerase (EC 2.7.7.19)	0.63	0.89	fig|1869229.4.6431	99
fig|1873126.6.peg.949	FIG 006163: hypothetical protein	0.49	0.93	No hit^b^	
fig|1873126.6.peg.1233	Cell division protein FtsQ	0.96	0.58	fig|1869229.4.1048	99
fig|1873126.6.peg.1635	FIG 00953621: hypothetical protein	0.79	0.64	No hit	
fig|1873126.6.peg.1843	Transcriptional regulator, LysR family	0.54	0.90	No hit	
fig|1873126.6.peg.1848	Tryptophan 2,3‐dioxygenase (EC 1.13.11.11)	0.44	0.52	fig|1869229.4.5262	99
fig|1873126.6.peg.1954	Purine‐cytosine permease	0.94	0.74	fig|1869229.4.5772	99
fig|1873126.6.peg.1993	Flagellar basal‐body rod modification protein FlgD	0.54	0.58	fig|1869229.4.5735	95
fig|1873126.6.peg.1999	Negative regulator of flagellin synthesis FlgM (anti‐sigma28)	1.11	0.88	fig|1869229.4.5729	93
fig|1873126.6.peg.2068	Gluconate utilization system Gnt‐I transcriptional repressor	0.5	0.72	fig|1869229.4.5645	99
fig|1873126.6.peg.2072	FIG 00964490: hypothetical protein	0.87	0.67	fig|1869229.4.5641	90
fig|1873126.6.peg.2543	Serine protease	0.45	0.52	No hit	
fig|1873126.6.peg.2702	L‐aminoadipate‐semialdehyde dehydrogenase large subunit (EC 1.2.1.31)	0.53	0.91	No hit	
fig|1873126.6.peg.2796	Protein of unknown function UPF0153	0.71	0.62	No hit	
fig|1873126.6.peg.2861	Uncharacterized MFS‐type transporter	0.45	1.00	No hit	
fig|1873126.6.peg.3237	Hypothetical protein	1.04	0.87	No hit	
fig|1873126.6.peg.3238	Hypothetical protein	1.15	0.55	No hit	
fig|1873126.6.peg.3512	Hypothetical protein	0.9	0.51	No hit	
fig|1873126.6.peg.3737	Aldehyde dehydrogenase (EC 1.2.1.3)	0.69	1.12	fig|1869229.4.4294	39
fig|1873126.6.peg.4115	Flagellar hook‐associated protein FlgL	0.54	0.91	No hit	
fig|1873126.6.peg.4117	Flagellar protein FlgJ([peptidoglycan hydrolase)	0.7	0.51	fig|1869229.4.6058	95
fig|1873126.6.peg.4220	Hypothetical protein	1.04	0.78	fig|1869229.4.5966	52
fig|1873126.6.peg.4221	Hypothetical protein	1.11	1.68	fig|1869229.4.5965	36
fig|1873126.6.peg.4786	Hypothetical protein	0.9	0.75	No hit	
fig|1873126.6.peg.5536	Uncharacterized MFS‐type transporter	0.78	0.65	No hit	
fig|1873126.6.peg.5637	Hypothetical protein	1.15	1.01	fig|1869229.4.3454	31
fig|1873126.6.peg.5795	Hypothetical protein	1.1	0.57	No hit	
fig|1873126.6.peg.6240	FIG 01212863: hypothetical protein	0.6	0.52	No hit	
fig|1873126.6.peg.1888	Type III effector HopPmaJ	0.5	N/A	fig|1869229.4.5302	93
fig|1873126.6.peg.6241	Candidate type III effector Hop protein		N/A	No hit	

N/A, not applicable as these potential T3SS effector proteins were not positively predicted by BPBAac tool.

a Predicted effector proteins were identified using BPBAac tools (Wang et al., 2011) on the over 500 effectors predicted by the T3SS effector prediction tool of Lower and Schneider (2009) (Lower).

b No hit indicates absence of homolog protein in strain S1E40 after BLASTp search at e‐value cutoff of 10^−5^.

### 3.6 Hypersensitivity and growth in M9 minimal medium

**Figure 6:**
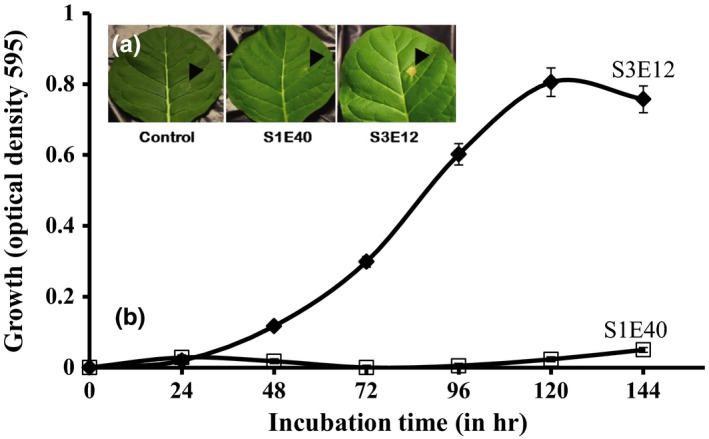
(a) Results of hypersensitivity reaction; and (b) growth curves of Pseudomonas sp. strains S3E12 and S1E40 performed in M9 minimal medium supplemented with 10 mM fructose (Shulte & Bonas, 1992). Note: strain S1E40 did not grow compared with S3E12 tobacco leaves as well as M9M medium

Strain S3E12 induced an hypersensitivity response after 24 hr while strain S1E40 did not (Figure 6a), suggesting that the latter can successful grow in plant cells than the former. This was confirmed in artificial M9M medium. Figure 6b shows the growth of strains S1E40 and S3E12 in M9M medium over a 6‐day period. After 24 hr of incubation, both strains showed little or no growth. Strain S3E12 showed a slow but steady growth after 48 hr up to 120 hr after which a decline was observed. In contrast, strain S1E40 did not show any significant growth during the same period.

To evaluate cell viability and validate growth of S3E12 in M9M, RNA was extracted and the expression of hopPmaJ effector (PATRIC identifier fig|1873126.6.peg.1888) and effector‐binding protein (PATRIC identifier fig|1873126.6.peg.2064) were assessed using SyBr Green real‐time chemistry. Figure S3 shows the fluorescence kinetics of the gene expression results. Positive detection of both genes in cDNA was achieved at threshold cycle of 27.01 and 27.99 for HopPmaJ (Figure S3) and effector‐binding protein (Figure S3), respectively. The expression of a candidate effector gene (PATRIC identifier fig|1873126.6.peg.6241) exhibited a similar Ct value (data not shown). All the negative control reactions did not exhibit any detectable fluorescence above the threshold fluorescence (TF).

## 4 DISCUSSION

This study sequenced and analyzed the genomes of two biological control strains (S1E40 and S3E12) of *Pseudomonas* to determine whether there are any differences in the T3SS organization and predicted T3SS effector proteins as well as other genome‐based features as these strains differ in their ability to induce HR on tobacco. Also, the taxonomic position of the strains was assessed using MLSA and genome‐based DNA–DNA homology analyses.

Multilocus phylogeny clustered the two strains consistently and distinctively with respect to known and validly described *Pseudomonas* species suggesting that these strains could be novel. Genome‐based DNA–DNA hybridization data are in agreement with phylogenetic data. Genome comparisons based on dDDH and ANIm showed values that are significantly below the cutoff threshold for species delineation, suggesting that these bacterial strains could be authentic novel species. A formal taxonomic study will provide a better insight.

The genomes of strains S3E12 and S1E40 were sequenced and assembled and different optimal k‐mer values identified for the two new strains. This suggests that the use of a wide range of k‐mer values is crucial for obtaining the best assemblies. In this study, we used k‐mer values from 50 to 150 obtaining the best assemblies with 38 (N_50_ = 206,686 bp) and 49 (N_50_ = 420,043 bp) contigs for S3E12 and S1E40, respectively. In contrast, using CLC software in parallel produced the best assemblies with 186 (N_50_ = 102,697 bp) and 126 (N_50_ = 290,793 bp) contigs for S1E40 and S3E12 (data not shown). One of the reasons why CLC showed high contig numbers compared to ABySS assembler could be the limitations in k‐mer options. CLC allows a maximum k‐mer value of 64 while k‐mer options of up to 250 can be implemented in the ABySS assembler.

Strains S3E12 and S1E40 induced different levels of HRs on tobacco and corn seedlings: strain S3E12 provoked intense HR at high bacterial populations, while strain S1E40 triggered little or no response in these plants. This suggests that S1E40 cannot grow under nutrient‐poor conditions. This hypothesis was validated using M9M medium, a medium reported to mimic nutrients availability in plant cells.

We analyzed the genomes for differences in the T3SS organization and predicted T3SS effector proteins (T3Es). The T3SSs are key factors for host cell interaction by many plant and animal bacteria leading to disease or HR response in a compatible host (Arnold et al., 2009; Bergeron et al., 2016; Goldberg et al., 2016; Petnicki‐Ocwieja et al., 2002). The T3SS represents multiprotein appendages or needle‐like structures that inject export T3Es inducing host reactions (Abby et al., 2016; Arnold et al., 2009; Goldberg et al., 2016). Advances in genomics and bioinformatics have led to the development of tools to scan genome data *in silico* for T3SSs and T3Es. This has provided significant insight to these important structures in microbe–host interactions. In our study, we implemented some of the T346Hunter prediction tool to identified T3SS clusters in the genomes of S1E40 and S3E12. While the two flagellar T3SS (F‐T3SS) cluster identified in both strains were conserved, the single nonflagellar T3SS (NF‐T3SS) cluster showed some variations. Most of the genes within this NF‐T3SS cluster showed similarity values of 84%–98%. However, one key gene, the HrpJ gatekeeper, had only 64% homology between the strains. In addition, within the NF‐T3SS cluster, strain S1E40 does not possess the hrcJ gene. The significance of these differences, especially the absence of HrcJ, is still to be elucidated. The NF‐T3SS evolved from flagella (Abby et al., 2016; Cornelis, 2006) and are complex protein machineries in Gram‐negative bacteria that deliver effector proteins into the cells of plant and animal hosts inducing pathogenesis (Abby & Rocha, 2012).

T3Es secreted through the T3SS appendages modulate the eukaryotic host cells leading to disease in compatible reactions. We used computational tools to identify potential effector proteins in the genomes of S1E40 and S3E12. The effector proteins predicted using a combination of bioinformatics tools differed significantly between the two strains. Sixteen protein effectors identified in the HR‐inducing strain S3E12 did not have homologs in the genome of the HR‐negative strain S3E40. Of these effectors, a CDS encoding for serine protease effector was found only in S3E12. Putative serine protease effectors of *Clavibacter michiganensis* are reported to induce a HR response in the apoplast of *Nicotiana* species (Lu, Hatsugai, Katagiri, Ishimaru, & Glazebrook, 2015). There are several pathogen‐encoded effectors that function as proteases secreted into host cells to modify the cytosol (Xia, 2004). In *Pseudomonas syringae*, however, a Hrp cysteine protease effector (HopPtoN) suppresses HR‐induced necrosis in plant host cells (Lopez‐Solanilla, Bronstein, Schneider, & Collmer, 2004). In our study, a CDs encoding for a putative cysteine protease (fig|1869229.4.peg.5802) was identified only in strain S1E40 but absent in S3E12. There is evidence that some bacterial effector proteins can suppress defense‐associated HR. For example, VirPphA is reported to block the Avr activity (Jackson et al., 1999) while AvrPtoB, a Pst DC3000 effector, was demonstrated to block the Avr activity domain within the AvrPtoB itself and the R gene‐dependent programmed cell death (Abramovitch, Kim, Chen, Dickman, & Martin, 2003). Future work on T3SS effectors proteins in strain S3E12 and S1E40 will focus on whether the proteases discussed above are partly involved in the HR‐inducing ability of strain S3E12 (serine protease) and S1E40 (cysteine protease).

Also, strain S3E12 has unique T1SS features which were not identified in strain S1E40. T1SSs are ubiquitous in a large number of Gram‐negative pathogenic phytobacteria mediating one‐step transport of substrates across both inner and outer membranes (Green & Mecsas, 2016; Pao, Paulsen, & Saier, 1998). This secretion system is comprised of a channel made of an ATP‐binding cassette (ABC) transporter, an outer membrane protein and membrane fusion protein (Delepelaire, 2004). The genome of strain S3E12 reveals CDSs encoding for a T1SS ATPase (LapB), a membrane fusion protein (LapC), a membrane bound c‐di‐GMP receptor (LapD), outer membrane protein (LapE), T1SS‐associated transglutaminase‐like cysteine proteinase (LapP), and T1SS secreted agglutinin repeats‐in‐toxins (RTX). RTX proteins containing agglutinin domains, like that of strain S3E12, might act as cell‐surface afrimbrial adhesins, facilitating cell–cell aggregation, formation of biofilm, and virulence for bacterial pathogens of plants and animals (Gottig, Garavaglia, Garofalo, Orellano, & Ottado, 2009; Guilhabert & Kirkpatrick, 2005; Roper et al., 2015; Voegel, Warren, Matsumoto, Igo, & Kirkpatrick, 2010). Toxigenic RTX proteins generally induce the lysis of host cells by forming pores in the host membranes (Benz et al., 1994; Gentschev,^2003^), thus causing significant tissue damage (Roper et al., 2015). In the *Pantoea stewartii* subsp. *stewartii* pathosystem, RTX‐like protein is reported to mediate host colonization by facilitating the development of water‐soaked lesions and leakage of cell content (Roper et al., 2015) leading to plant damage.

While T1SSs are not often considered major factors in pathogenesis, many bacterial phytopathogens use these systems for extracellular secretion of virulence factors. Zhang, Bak, Heid, and Geider (1999) showed that T1SS in *Erwinia amylovora* is used to secrete virulence‐associated metalloprotease (PrtA), a protein necessary for the colonization of apple leaves. T1SSs, in the *Pseudomonas syringae*‐cherry fruit pathosystem, are reported to be involved in the secretion of pore‐forming syringopeptin and syringomycin phytotoxins during colonization leading to extensive tissue necrosis (Carezzano et al., 2017; Hutchison & Gross, 1997; Quigley, Mo, & Gross, 1993; Scholz‐Schroeder, Hutchison, Grgurina, & Gross, 2001).

In addition, to transport of pathogenesis/virulence‐related proteins, T1SSs are known to have a defensive role in protecting the bacteria from plant antimicrobial secondary compounds such as phytoalexins and phytoanticipins (Vanetten, Mansfield, Bailey, & Farmer, 1994). Both strains (S1E40 and S3E12) have at least six CDSs encoding for TolC mediated multidrug efflux system which have been implicated in the defense of *Xylella fastidiosa* (Reddy, Reddy, Hopkins, & Gabriel, 2007), *Dickeya dadanti* (Barabote et al., 2003), and *E. amylovora* (Burse, Weingart, & Ullrich, 2004) for actively exporting antimicrobial compounds produced by plants.

Differences in the genomes of the two strains also included a number of CDSs involved in Pel polysaccharide (Pel) production and alkylphosphonate utilization. In *Pseudomonas aeruginosa*, Pel production is reported to serve as intercellular adhesin for biofilm formation and maintenance (Colvin et al., 2013). Bacterial cells in the biofilm are coated in extracellular polysaccharides (EPS) protecting cells from host defenses and antimicrobial compounds (Stewart & Costerton, 2001; Stoodley, Sauer, Davies, & Costerton, 2002). Strain S1E40 has CDSs of all seven‐gene operon (*pel*A‐*pel*G) required for Pel biosynthesis, and their presence in the genome was confirmed by PCR amplification (data not shown). None of the *pel* genes or the seven‐gene operon were identified in the genome of strain S3E12, suggesting that biofilm formation in these two strains is modulated differently. Strain S3E12 has a CDS encoding for levansucrase (EC 2.4.1.10), a EPS reported to be partly involved in biofilm formation in *P. syringae* (Hettwer, Gross, & Rudolph, 1995) and *E. amylovora* (Bogs & Geider, 2000).

In addition, both strains were shown to have four CDSs involved in alkylphosphonate utilization: ATP‐binding protein PhnN, alkylphosphonate utilization operon proteins PhnA, PhnB, and PhnO. However, strain S1E40 has a unique cluster of 8 CDSs encoding for alkylphosphonate utilization. These CDSs encode for four proteins PhnG, PhnH, PhnI, and PhnJ with two phosphonates transport ATP‐binding proteins (PhnK and PhnL) and a transcriptional regulator (PhnF). Also, a CDS encoding for a metal‐dependent hydrolase involved in phosphonate metabolism and a novel secreted alkaline phosphatase were identified in the genome of S1E40 but not in S3E12. Under natural conditions, bacteria play a significant role in degradation of phosphonates (Huang, Su, & Xu, 2005; Nowack, 2003), and because of the exposure to natural phosphonates, bacteria have evolved to utilize phosphonates as nutrient sources. The presence of the complete operon in S1E40 suggests that this bacterial strain can effectively use phosphonates as a potential source of phosphorus for growth.

This study assessed the taxonomic position of two new *Pseudomonas* strains based on 16S rRNA, MLSA, and genome‐based DNA homology and concludes that there is ample evidence to perform a detailed analysis for description of these strains as novel species. In addition, a detailed comparison of the genomes of these strains revealed differences in the T3SSs as well as predicted T3Es in the HR‐inducing S3E12 and the HR‐negative strain S1E40. Data from this study suggest that the T3SSs organization of the two strains differ. Also, strain S3E12 exhibited sixteen T3Es not present in S1E40 including a serine protease effector reported to incite HR on *Nicotiana* species by *C. michiganensis*. In contrast, strain S1E40 possesses a unique CDS encoding for a cysteine protease, a protein capable of suppressing HR responses. Future work using mutants is being planned to determine the potential role of these unique serine protease (S3E12) and cysteine protease (S1E40) in inducing or suppressing HR on tobacco seedlings. In addition, strain S3E12 has unique T1SS features such as T1SS secreted agglutinin RTX toxins, a group of cytolysins and cytotoxins produced by bacteria (Lally, Hill, Kieba, & Korostoff, 1999). In contrast, strain S1E40 has unique CDSs encoding for Pel production. This seven‐gene operon was not identified in strain S3E12, suggesting that *pel* polysaccharide might not be involved in biofilm formation mechanism. The data presented here provide an insight of the functional organization of the genomes of strains S1E40 and S3E12 as well as better understanding of these phytobacteria.

## CONFLICT OF INTEREST

None declared.
