# Supplementary material for: Genome analysis of two novel Pseudomonas strains exhibiting differential hypersensitivity reactions on tobacco seedlings reveals differences in nonflagellar T3SS organization and predicted effector proteins
Source: Microbiologyopen. 2018 Feb 21;7(2):e00553. doi: 10.1002/mbo3.553 (PMC5911992; doi:10.1002/mbo3.553)
Supplement: Supplementary file 1 [file MBO3-7-na-s001.pdf]

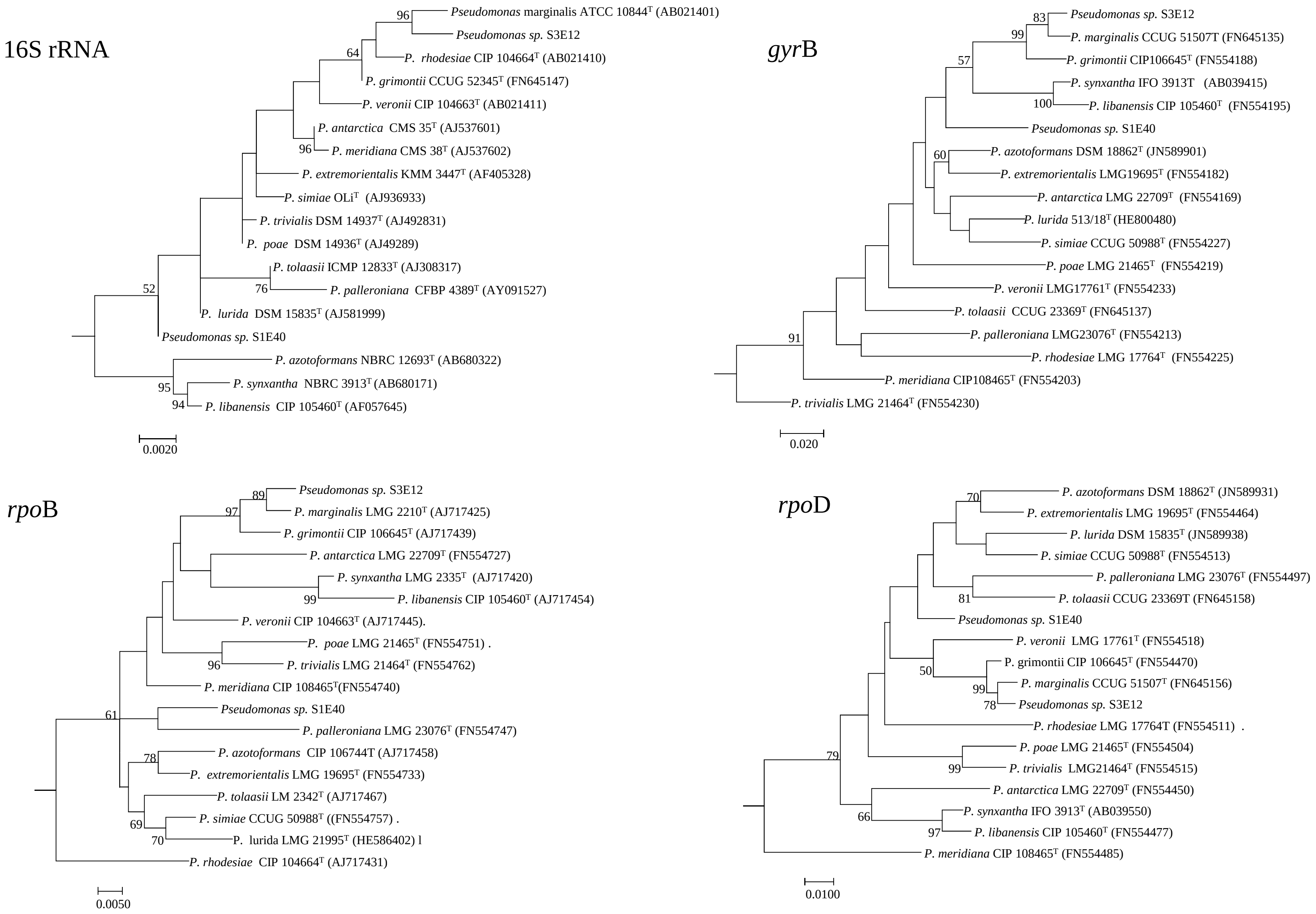

Figure S1. Single gene phylogenetic trees inferred by maximum-likelihood (ML) of *Pseudomonas sp.* strains S1E40 and S3E12 with type strains of close lineages using the general time reversible substitution model. A, 16S rRNA; B, *gyr*B; C, *rpo*B; and D, *rpo*D; ML trees were reconstructed for each gene sequences with 1000 bootstrap replicates. Bootstrap values higher than 50 are shown on nodes. *Pseudomonas aeruginosa* LMG1242^{T} was used as outgroup.
