# Supplementary material for: Genome analysis of two novel Pseudomonas strains exhibiting differential hypersensitivity reactions on tobacco seedlings reveals differences in nonflagellar T3SS organization and predicted effector proteins
Source: Microbiologyopen. 2018 Feb 21;7(2):e00553. doi: 10.1002/mbo3.553 (PMC5911992; doi:10.1002/mbo3.553)
Supplement: Supplementary file 2 [file MBO3-7-na-s002.pdf]

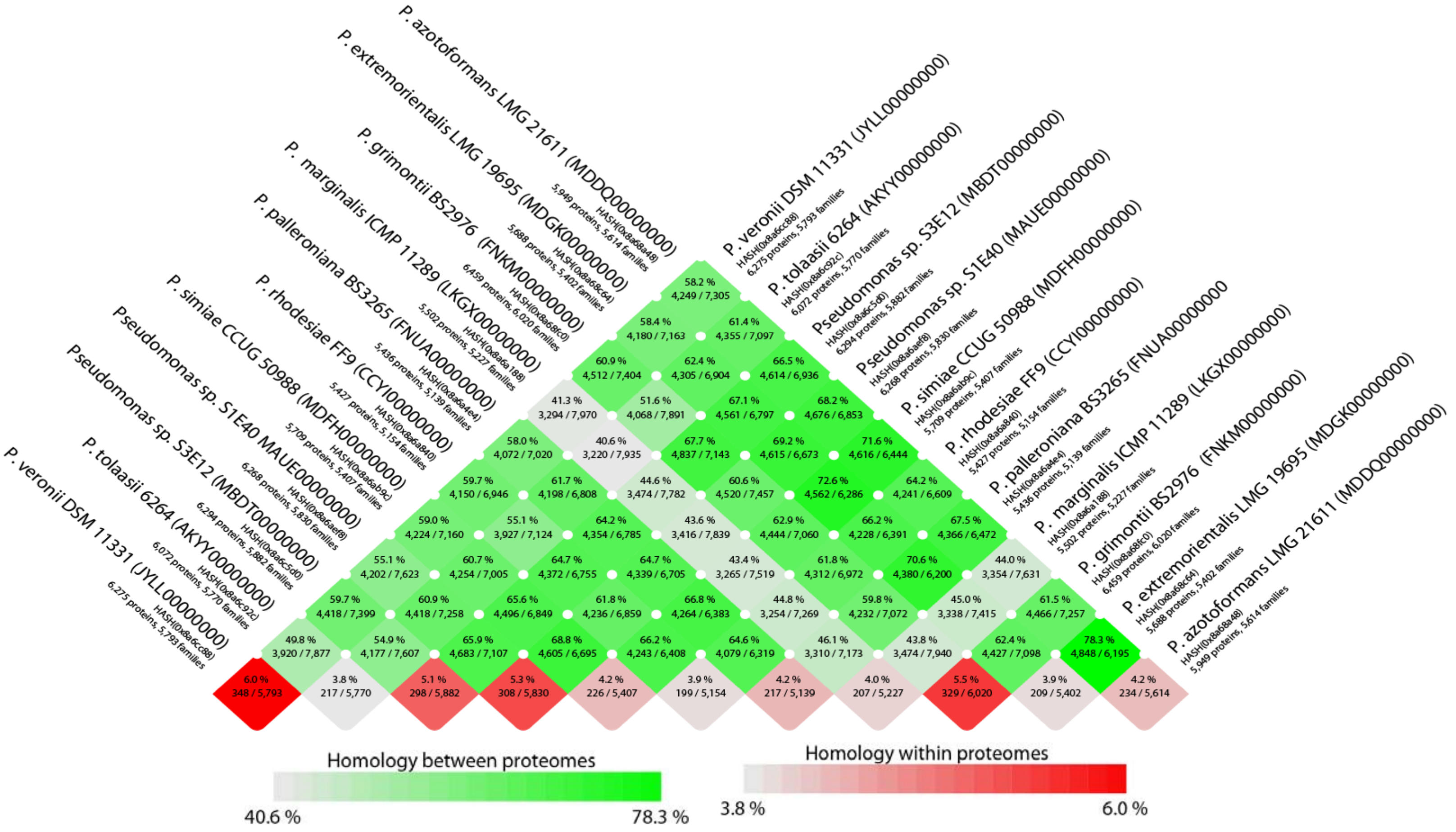

Figure S2. BLAST matrix of proteomes of the two new *Pseudomonas sp.* strains (S1E40 and S3E12) and their close lineages based on all against all protein comparison to define homologs. A hit is considered significant if 50%/50% (identity/length coverage) requirement between-proteomes is met. Paralogs (internal homology) are proteins within a genome matching the same 50–50 rule. BLAST matrix was performed using CMG-Biotools (Vesth *et al*., 2013).
