# Supplementary material for: Genome analysis of two novel Pseudomonas strains exhibiting differential hypersensitivity reactions on tobacco seedlings reveals differences in nonflagellar T3SS organization and predicted effector proteins
Source: Microbiologyopen. 2018 Feb 21;7(2):e00553. doi: 10.1002/mbo3.553 (PMC5911992; doi:10.1002/mbo3.553)
Supplement: Supplementary file 3 [file MBO3-7-na-s003.pdf]

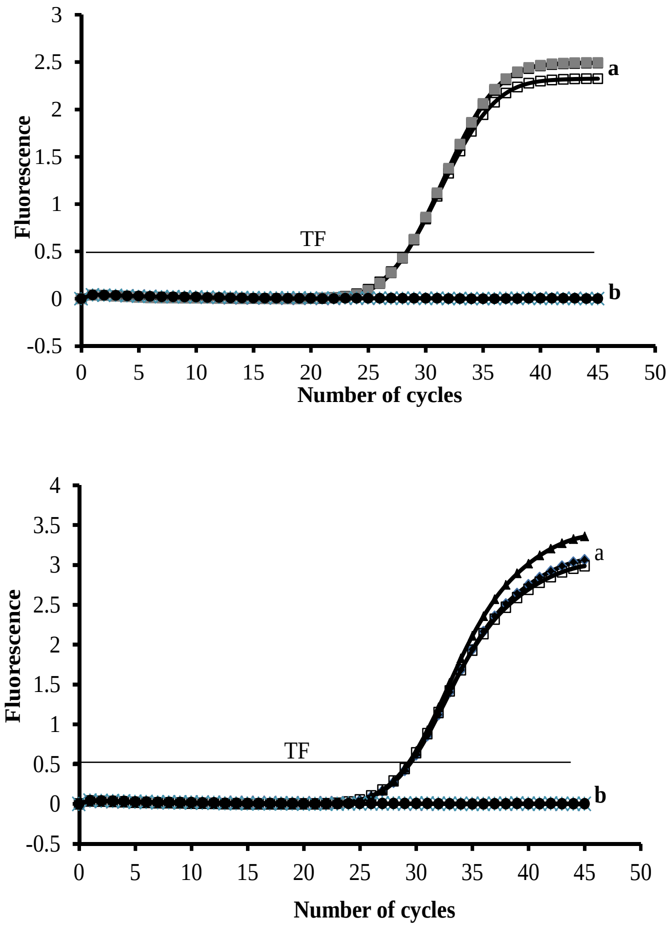


Figure S3. SyBr Green real-time PCR detection of expressed **(A)** Type III effector protein hopPmaJ (PATRIC gene identifier fig|1873126.6.peg.1888) and **(B)** a transcription activator, effector binding protein (PATRIC identifier fig|1873126.6.peg.2064) in cultures of strain S3E12 grown in M9 minimal liquid medium after 6 days: (a) fluorescence kinetics of targeted gene and (b) negative control reaction. Data show similar expression of the two target genes based on the identical Ct values of about 27.0 for hopPmaJ and 27.9 effector binding protein at a preset threshold fluorescence  (TF) of 0.5. SyBr Green real-time assay was performed on triplicate samples and repeated twice.
