# Supplementary material for: Genome analysis of two novel Pseudomonas strains exhibiting differential hypersensitivity reactions on tobacco seedlings reveals differences in nonflagellar T3SS organization and predicted effector proteins
Source: Microbiologyopen. 2018 Feb 21;7(2):e00553. doi: 10.1002/mbo3.553 (PMC5911992; doi:10.1002/mbo3.553)
Supplement: Supplementary file 4 [file MBO3-7-na-s004.docx]

| Table S1. Statistics of the genomes used to determine digital DNA-DNA hybridization homology and MuMer-based average nucleotide identity. | | | | | | | | |
| --- | --- | --- | --- | --- | --- | --- | --- | --- |
| *Pseudomonas* species | Size (Mb) | GC% | # of proteins | rRNA | tRNA | Other RNA | Gene | Pseudogene |
| *P. palleroniana* BS3265 (FNUA00000000) | 6.15 | 60.5 | 5,417 | 20 | 70 | 102 | 5,659 | 50 |
| *P. tolaasii* 6264 (AKYY00000000) | 6.23 | 61.0 | 5,234 | 0 | 54 | 1 | 5,965 | 671 |
| *P. simiae* CCUG 50988T (MDFH00000000) | 6.29 | 60.3 | 5,585 | 17 | 63 | 4 | 5,724 | 55 |
| *P. azotoformans* LMG 21611^T^ (MDDQ00000000) | 6.72 | 61.0 | 5,888 | 15 | 71 | 4 | 6,022 | 44 |
| *P. extremorientalis* LMG 19695^T^ (MDGK00000000) | 6.35 | 60.9 | 5,578 | 6 | 46 | 4 | 5,706 | 72 |
| *P. rhodesiae* FF9 (CCYI00000000) | 6.05 | 60.4 | 5,282 | 4 | 58 | 1 | 5,414 | 69 |
| *P. veronii* DSM 11331 (JYLL00000000) | 7.00 | 60.7 | 6,049 | 7 | 60 | 1 | 6,226 | 109 |
| *P. grimontii* BS2976 (FNKM00000000) | 7.11 | 60.1 | 6,343 | 22 | 71 | 114 | 6,703 | 153 |
| *P. marginalis* ICMP 11289 (LKGX00000000) | 6.09 | 59.2 | 5,316 | 6 | 60 | 4 | 5,486 | 100 |
| *Pseudomonas sp.* S3E12 (MBDT00000000) | 7.06 | 60.9 | 6,127 | 34 | 79 | 4 | 6,337 | 93 |
| *Pseudomonas sp.* S1E40 (MAUE00000000) | 6.98 | 61.6 | 6,148 | 12 | 61 | 4 | 6,289 | 64 |
